# 

**Published:** 1883-06

**Authors:** 


### INDEX TO VOLUME XLVI.

PAGE.

A.

Abdominal Section, 25 Cases of, J. Ewing Mears.......... 66
Abdominal Section, 200 Cases of, Lanson Tait.... ....... 407
Action of Quinine on the Heart. 183
Agaric in Phthisical Night-Sweats.................. 78
Albuminuria, Treatise on, W. H. Dickinson, Review ......... 617
American Medical Association, Announcement ........... 663
American Medical Association Journal, Announcement..... 666
Andrews, E., Stockading a Loose Cartilage............. 32
Antiseptic Treatment of Typhoid Fever............. 44
Annals of Anatomy and Surgery, Personal Reflections... 105
Artistic Anatomy, S. V. Clevenger ........................ 113
Argentine Republic....... 188
Army Medical Board ........ 329
Autopsy of Gambetta.......... 186
Aspiration of the Pericardium. 44
Auscultation and Percussion, Review ..................... 416
Anatomical Technology, B. G. Wilder and S. H. Gage, Review..... ................ 173

B.

Bacillus of Glanders.......... 396
Banquet at the Academy of Medicine.................... 667
Bartholow, Roberts, Treatment of Spinal Diseases.......... 534
Basilar Meningitis, Non-Tubercular...................... 432

PAGE.

Barker, Fordyce.............. 332
Baker, Henry B., Government Protection Needed in Prevention of Contagious Diseases.. 102
Bates, M. E., Item............ 672
Baxter, A. J , Item......... ... 175
Bennett, E. R., Report of Two Cases of Hydroa ........... 476
Belfield, W. T., Notice, Cartright Course of Lectures..... 111
Belfield, W. T., Item ......... 662
Belfield, W. T., Exhibition at Chicago Med. Society, April 16, 1883.................... 603
Biliousness, George Harley.... 318
Blatta Orientalis, Treatment of Hydrops by ................ 419
Book Notices................. 110
Box Theory.................. 188
Books and Pamphlets Received. ........................246, 416
Bogue, R. G., Item ........... 246
Bogue, R. G., New Operation for Varicocele .............. 382
Bogue, R. G., Cases from Practice.......... ........... 470
Brower, D. R., and F. H. Martin, Tumor in Motor Area of Cortex .................... 21
Bright's Disease, Clinical Diagnosis of, C. W. Purdy....... 1
Brain, Weight of in Mental Diseases .................. 187
Brain, Changes of in Infectious Diseases ... ... .. ........ 431
Brain, Abscess of.......... .... 424
Bright's Disease, Pathology of, C. W. Purdy................ 352
Bristowe, J. S., Remarks on the Functional Vomiting of Hysteria...................... 648
Bubo, Treatment of, G. F. Lydston ..................... 478

PAGE.

C.

Casselberry, W. E., Cough of Remote Origin.............. 570
Cases From Practice, R. G. Bogue.................... 470
Cauterization of the Clitoris... 671
Cancer of the Peritoneum..... 42
Carpenter, A. M., Case of Locomotor Ataxia............... 28
Cerebro Spinal Syphilis....... 632
Chicago Medical Colleges.... 223
Clemenceau, Dr.............. 188
Clinics..........112, 224, 336, 448
Clinical Lecture, David W. Cheever.................. 299
Clevenger, S. V., Artistic Anatomy and the Sciences Useful to the Artist................ 113
Correction.................. 222
....................169, 408, 614
Cook County Hospital..445, 665, 175
Colleges of the World......... 187
Cough of Remote Origin, W. E. Casselberry............. 570
Critchett, Dr. George, Death of. 45
Craver, Dr. S. B., Item....... 335
Cushing, Clinton, What Are the Conditions that Justify Oöphorectomy................. 525
Columbus Medical College.... 668
Chronic Polygastritis......... 43
Chlorinated Soda............. 335
Cheever, David N., Clinical Lecture.................... 299
Chlorate of Potassium, Are Ordinary Doses Ever Poisonous? E. Ingals.................... 245
Chiari, The Pathologist....... 524
Chicago Medical Society...602, 500
Charcot & Loomis, Clinical Lectures on the Diseases of Old Age.................. 619
Chicago Pathological Society.. .......................415, 602

D.

Dana, C. L., Absorption of Nutrient Enemata............. 324
Danforth, I. N., Clinic at St. Luke's................... 415
Dana, C. L., Delusions in Regard to the Oyster......... 315
Davis, N. S., Pneumonia in Chicago in 1882............. 449
Davis, E. P., Report, Hospital.. 665
Dawson, B. F., Item.......... 335
Davis, E. P., Suppurative Otitis Media Pyæmia, Case in Hospital...................... 392

PAGE.

Deflected Septum Narium, Moses Gunn................ 223
Dermoid Cyst of Ovary....... 517
Dental Infirmary.............. 328
Dobbins, A. E., Item.......... 335
Doctorate Address, Moses Gunn 337
Dorland, Walter L., Item...... 672
Double Placenta.............. 639
Double Urethra in Man....... 33
Dickinson, W. H., Treatise on Albuminuria, Review...... 617
Diseases of Women, Clinical Lecture by T. Gaillard Thomas...................... 542
Diseases of Old Age, Lectures on, by Charcot & Loomis.... 619
Dissolution of Fish Bones in Throat..................... 619
Diseases of Children, Edward Henoch, Review........... 618
Dover, Dr., Sketch of.......... 326
Diphtheria Treated by Homœopaths..................... 524
Dyas W. Godfrey, What is Disease?..................... 561
Dynamite in Medicine....... 45
Dysentery, Treatment of....... 325

E.

Echinococcus in Right Plural Cavity..................... 638
Editors, Society of American. 446
Editorial..................514, 620
Electrizations, Cephalic and Spinal. Therapeutic Value, G. H. Hughes................. 267
Enteric Fever, Management of, J. C. Wilson.............. 257
Enemata, Nutrient Absorption of, C. L. Dana............. 324
Enchondroma of the Parotid... 637
Entropium and Trichiasis, Remarks on, F. C. Hotz........ 640
Epigastric Pains in Hysteria, Electrical Treatment of...... 188
Explosive Mixtures in Medicine....................... 613
Exciting Cause of Hysteria and Hystro Epilepsy, Graily Hewitt......................... 189

F.

Faculty Meeting, Woman's Medical College............... 672
Female Medical Education.... 332
Felt Jackets Instead of Plaster. 619
Fissure of the Anus in a Child. 185
Fistula Ani Treated by Elastic Ligature.................. 42

PAGE.
Fistula, Traumatic Recto Vesical.......... 330
Fitch, T. Davis, Item.......... 672
Flint, Austin, The Self-Limited Duration of Pulmonary Phthisis.......... 81
Frontiers of Madness.......... 312
Foster, A. H., Scarlet Fever in One Family.......... 243

G.

Gage, S. H., Anatomical Technology.......... 173
General Paralysis. P. Zenner... 204
Gastroscopia and Æsophagascopia.......... 43
Gambetta, Necropsy of.......... 320
Gynæcology, Manual of, Hart & Barbor, Review.......... 616
Glanders, Bacillus of.......... 396
U. S. Pharmacopœia.......... 374
Government Protection Needed, Henry B. Baker.......... 102
Griswold, Observations on the Tampon in the Hæmorrhage of Abortion.......... 88
Granular Conjunctivitis, Treatment.......... 672
Graham, D. W., Item.......... 672
Gunn, Moses, Doctorate Address 337

H.

Hæmatoma of the Pancreas.... 630
Harvard, Chair of Anatomy at. 335
Harley, George, On Diseases of the Liver.......... 318
Hart & Barbour, Manual of Gynæcology, Review.......... 616
Hardaway, W. A., Vaccination, Essentials of.......... 175
Health, Board of, Illinois...332 447
Heart, Valvular Disease of, Sansom.......... 247
Heart Puncture and Heart Suture, John B. Roberts.......... 305
Hernia, Obturator Incarcerata. 634
Hennoch, Edward, Lectures on Diseases of Children.......... 618
Henrotin, F., Item.......... 175
Hewitt, Graily, Cause of Hysteria and Hystero Epilepsy... 189
Heitzmann, C., Microscopical Morphology of the Animal Body in Health and Disease 170
Hereditary and Pathological Aspect of Vice, G. F. Lydston 131
Homœopathic Hospital Staff... 662
Hotz, F. C., Remarks on 177 Cases of Entropium and Trichiasis.......... 640

PAGE.
Hughes, G. H., Therapeutic Value of Cephalic and Spinal Electrizations.......... 267
Hutchinson, James C., Contributions to Orthopœdic Surgery.......... 511
Hyde, Jumes Nevins, Practical Treatise on Diseases of the Skin, Review.......... 509
Hydroa, Two Cases of, E. R. Bennett.......... 476
Hysterical Mania, W. B. Kesteven.......... 309
Hypnotism, Case of.......... 36

I.

Iodine in Erysipelas and Small-Pox.......... 80
Illustrated Quarterly of Medicine and Surgery.......... 331
Illustrated Medicine and Surgery.......... 334
Illinois State Board of Health. 581
Ingals, E. Fletcher, Lady Physicians.......... 390
Ingals, E. Fletcher, Notice.. 111
Ingals, E., Infection of Scarlet Fever.......... 31
Ingals, E., Is Chlorate of Potassium in Ordinary Doses Ever Poisonous?.......... 245
Infection, Criminal Spread of.. 327
Iodoform, To Disguise the Odor of.......... 330
Iodoform in Diphtheria.......... 323
Items..........111, 221 328

J.

Jeancon, I. A., Pathological Anatomy.......... 174
Jenks, Edw. W., Modified Listerism in Ovariotomy.......... 434
Jewell, S. J. Dr., Improved Health.......... 335

K.

Keen, W. W., Report on Xanthic Oxide Calculus.......... 217
King, A. F. A., Manual of Obstetrics, Review.......... 171
Kesteven, W. R., Hysterical Mania.......... 309

L.

Lagorio, A., Hypodermic Treatment of Syphilis.......... 459
Lady Physicians, E. Fletcher Ingals.......... 390

PAGE.
Laryngeal Spasm in a Case of Locomotor Ataxy........... 523
Larynx, Traumatic Stenosis of. 331
Las Vegas, Medical Society of New Mexico................ 110
Leprosy, Importation of....... 335
Leucoplakia Buccalis......... 185
Leiters' Tubes as Regulators of Temperature............... 186
Leprosy in Massachusetts...... 580
Locomotor Ataxia, Case, Recovery, A. M. Carpenter......... 28
Locomotor Ataxia, Dry Cupping and Rest in Treatment, H. M. Lyman............... 98
Locomotor Ataxy............ 661
Loomis, Alfred L. and J. M. Charcot, Diseases of Old Age. 619
Lowe, John, Puerperal Fever, Its Treatment and Prevention 93
Lydston, G. Frank, The Treatment of Bubo............. 478
Lydston, G. F., Contribution to the Hereditary and Pathological Aspect of Vice.......... 131
Lyman, H. M., Dry Cupping and Rest in the Treatment of Locomotor Ataxia........... 98

M.

MacDonnell, R. L., Therapeutic Notes.................... 73
Malarial Fevers.............. 40
Martin, F. H., and D. R. Brower, Tumor in Motor Area of Cortex......................... 21
Malaria in Flower-Pots........ 639
Maryland Medical Journal.... 672
Materia Medica of the Sixteenth Century.................. 163
Mayer, H. M., Item........... 266
Mears, J. Ewing, 25 Cases of Abdominal Section.......... 66
Medical Examination by State Boards.................... 620
Medical Societies............. 223
Mergler, M. J., Item.......... 672
Micrococci, Vaccinal.......... 333
Michigan State Board of Health .................... .164, 607
Microbium of Blennorrhagic Pus........................ 521
Microscopical Morphology of the Animal Body........... 170
Micro Organism in Yellow Fever..................... 580
Moluscum Fibrosum.......... 635
Montgomery, W. T., Notice.... 97
Mortality of the Globe........ 499

PAGE.
Munde, P. F., Secondary Puerperal Hæmorrhage......... 275
Mullein Plant in the Treatment of Pulmonary Consumption.. 322
Mudd, S. A., Item, Death of.... 330
Myo-Fibroma of Uterus, Extirpation Per Vaginum....... 629

N.

Nerve-Stretching, Results of... 107
New York Letter.............. 408
New Symptom of Cerebral Disease......................... 219
Notes on Therapeutics. R. L. Mac Donnell................ 73

O.

Obituary, D. O. Farrand....... 433
Obstetrics, Manual of, A. F. A. King, Review.............. 171
Official List of Changes in U. S. M. H. S.................... 221
Ogle County Medical Society.. 508
Oliver, Dr. E. K., Item........ 335
Oöphorectomy, What Are the Conditions That Justify It? Clinton Cushing............ 525
Ophthalmological Clinic of Dr. Mooren, of Dusseldorf..... 34
Orthopœdic Surgery, Hutchinson, Review................ 511
Oriental Judgment in Malpractice...................... 671
Ossification of the Choroid..... 420
Otitis, Suppuration Caused by Tamponment of the Nasal Fossæ.................... 41
Otitis Media Purulenta, E. P. Davis...................... 392
Ovariotomy With Modified Listerism, Edward W. Jenks.... 434
Ovariotomy, Accidents in...... 628
Owens, J. E., Item............ 672
Oxide of Zinc Poisoning...... 522
Oyster; the Delusions in Regard to, C. L. Dana............... 315

P.

Pachymeningitis.............. 44
Pallen, M. A. Dr., Item....... 335
Paralysis from Injection of Ether...................... 432
Park, Roswell, Storage of Electricity.................... 148
Parkes, C. T., Case of Complete Vertical Dislocation of the Patella.................... 387

PAGE.
Patella, Dislocation of, Chas. T. Parkes.... 387
Papilloma of Bladder.... 638
Pain in Right Iliac Region.... 636
Pathological Anatomy, J. A. Jeancon.... 174
Peritoneal Cancer.... 42
Pettijohn, Item.... 246
Peritonitis, Pyæmic.... 635
Phthisis, Self-Limited Duration of, Austin Flint.... 81
Physicians and Surgeons College of—Changes in.... 447
Physicians and Surgeons College of, Commencement.... 405
Pharmacopœia U. S., C. E. Clacius.... 374
Philadelphia Hospital for Skin Diseases.... 669
Pharmacopœia of the United States, Review.... 172
Pneumonia in Chicago in 1882, N. S. Davis.... 449
Polyorchism.... 45
Polio-Myelitis Anterior.... 37
Policlinic, New York.... 332
Post-Partum Hæmorrhage.... 520
Powers, Sam'l A., Variola, Review.... 512
Pott's Disease.... 178
Polydipsia, Case of, D. D Marr 161
Pregnancy with Unperforated Hymen.... 45
Prize, the Hammond.... 333
Prolapsus Recti, New Operation.... 637
Prolapse of Stomach through the Umbilicus.... 636
Psoriasis of Skin and Mucous Membranes.... 39
Pseudo Bacillus of Tuberculosis, H. D. Schmidt.... 225
Puerperal Fever, Its Treatment and Prevention, John Lowe.. 93
Purdy, Chas. W., Clinical Diagnosis of Bright's Disease ... 1
Puerperal Hæmorrhage, Secondary, P. F. Munde.... 275
Purdy, C. W., Pathology of Bright's Disease.... 352
Pulsation of Veins.... 577
Purpura Rheumatica.... 187

Q.

Quine, W. E., Item.... 606
Quassine and Its Uses.... 522

R.

Retinitis, Unilateral Albuminar.... 630

PAGE.
Retention of a Fœtal Head in the Uterus for Forty Days... 633
Retention of Placenta.... 637
Retention of the Placenta for Two Years.... 433
Reviews and Book Notices. 170, 416, 509, 615
Repetition of Doses, A. A. Smith.... 291
Reception to Drs. Atlee and Stone.... 334
Rheumatic Ostitis.... 183
Roberts, John B., Heart-Puncture and Heart Suture.... 305
Rosencrans, H., Letter from.... 614
Rosencrans, H., Letter from.... 169
Rush College, Forty-first Commencement.... 397
Russian Baths.... 626
Rush Medical College, Item... 447

S.

Sansom, A. E., Valvular Disease of Heart.... 247
Sarcoma of the Brain.... 38
Sarcoma of the Tonsils Cured by Iodoform.... 184
Salivary Fistula of the Canal of Steno.... 177
Scarlet Fever, Infection of, E. Ingals.... 31
Scarlet Fever in One Family, A. H. Foster.... 243
Schmidt, H. D., Pseudo Bacillus of Tuberculosis.... 225
Schmidt, H. D., Supplementary Paper.... 111
Selections, 189, 247, 434, 525, 640
Selections, Bradshaw Lecture on Some New and Rare Diseases, Sir James Paget.... 46
Seguin Dr., Improvement in Health.... 334
Seguin, Dr. E. C., Notice.... 45
Secreting Epithelium of the Kidneys in the Frog.... 184
Secondary Batteries and the So-called Storage of Electricity, Roswell Park.... 148
Skin, Diseases of, J. N Hyde.. 509
Skepticism in Medicine.... 580
Smith, A. A., Frequent Repetition of Doses.... 291
Society Reports, 164, 414, 500, 581
Society, Chicago Pathological. 415
Some Points in Generation.... 523
Stockading a Loose Cartilage to Fix it for Operation.... 32
Spinal Diseases, Treatment of, Roberts, Bartholow.... 534

PAGE.
Specimen of Xanthic Oxide Calculus, Report of W. W. Keen .......... 217
Surgery á la Wagner.......... 427
Stille, Alfred.......... 332
Sleater, S. W., Death of.......... 447
Strychnia Poisoning Cured by Chloral Hydrate.......... 430
Strictures of the Larynx .......... 423
Stenosis Uteri, O. Stroinski.......... 394
Stroinski, O, Translations.......... 33
Strychnine, Action of on the Heart.......... 188
Stomach, Transposition of.......... 182
Syphilitic Tumor of the Cortex in Motor Area. Case, Brower and Martin.......... 21
Syphilis of the Pharynx.......... 430
Syphilis and Dementia Paralytica.......... 428
Syphilis of the Spinal Cord.......... 426
Syphilis, Hypodermic Treatment of, A. Lagorio.......... 459
Syphilitic Erythema Multiforme.......... 638
Syphilitis and Rachitis, Hereditary.......... 631
Syphilitic Keratitis, Case.......... 179
Syphilitic Gumma of the Liver 176

T.

Tampon in Hæmorrhage of Abortion, R. W. Griswold.......... 88
Tetanus in Lying-in Women.......... 428
Thomas, T. Gailliard, Clinical Lecture on the Diseases of Women.......... 542
Tic de Salaam.......... 638
Tit but not Tat.......... 335
Translations from Foreign Exchanges, O. Stroinski....176, 420, 517, 626
Transplantation of Muscles.......... 639
Treatise on Therapeutics, H. C. Wood.......... 615
Tri-State Medical Society, Announcement of.......... 414
Tumor of the Hard Palate.......... 433
Tucker, J. J., Thoughts on Death of Richard Wagner.......... 241
Typhoid Fever, Treatment of. 520

PAGE.

U.

Ulcer of the Stomach.......... 633
Umbilical Cancer.......... 429
Uterine Subserous Fibroid.......... 425

V.

Vaccination, Essentials of, W. A. Hardaway.......... 175
Varicocele, New Operation For, R. G. Bogue.......... 382
Variola Hæmorrhagic, Etiology of.......... 181
Variola, Review, Samuel A. Power.......... 512
Venomous Apparatus of the Scorpion.......... 180
Vision, Temporary Loss by Papillitis.......... 181
Vital Statistics for February.......... 413
Vital Statistics for March.......... 524
Virchow, Prof., Attack of Nephritis.......... 45
Vital Statistics for April.......... 671
Vomiting of Hysteria, Remarks on, J. S. Bristowe.......... 648

W.

Wagner, Richard, Thoughts on Death of, J. I. Tucker.......... 241
Watson, Sir Thomas, Item.......... 513
Watson, Thomas, Obituary.......... 168
What is Disease? W. G. Dyas.......... 561
Wilder, B. G., Anatomical Technology.......... 173
Wilson, J. C., Management of Enteric Fever.......... 257
Woman's Medical College, Item. 447
Wood, H. C, Treatise on Therapeutics, Review.......... 615
Woman's College, Thirteenth Commencement.......... 400

Z.

Zenner, Philip, General Paralysis.......... 204

